# Integrated Analysis of RNA-Binding Proteins in Glioma

**DOI:** 10.3390/cancers12040892

**Published:** 2020-04-07

**Authors:** Zhixing Wang, Wanjun Tang, Jiangang Yuan, Boqin Qiang, Wei Han, Xiaozhong Peng

**Affiliations:** 1 State Key Laboratory of Medical Molecular Biology, Department of Molecular Biology and Biochemistry, Institute of Basic Medical Sciences, Medical Primate Research Center, Neuroscience Center, Chinese Academy of Medical Sciences, School of Basic Medicine, Peking Union Medical College, Beijing 100730, China; 2 Institute of Medical Biology, Chinese Academy of Medical Sciences, Peking Union Medical College, Kunming 650031, China

**Keywords:** glioma, RNA-binding protein, non-canonical RBP, WGCNAs, survival, prognosis, RIP-seq

## Abstract

RNA-binding proteins (RBPs) play important roles in many cancer types. However, RBPs have not been thoroughly and systematically studied in gliomas. Global analysis of the functional impact of RBPs will provide a better understanding of gliomagenesis and new insights into glioma therapy. In this study, we integrated a list of the human RBPs from six sources—Gerstberger, SONAR, Gene Ontology project, Poly(A) binding protein, CARIC, and XRNAX—which covered 4127 proteins with RNA-binding activity. The RNA sequencing data were downloaded from The Cancer Genome Atlas (TCGA) (*n* = 699) and Chinese Glioma Genome Atlas (CGGA) (*n* = 325 + 693). We examined the differentially expressed genes (DEGs) using the R package DESeq2, and constructed a weighted gene co-expression network analysis (WGCNA) of RBPs. Furthermore, survival analysis was also performed based on the univariate and multivariate Cox proportional hazards regression models. In the WGCNA analysis, we identified a key module involved in the overall survival (OS) of glioblastomas. Survival analysis revealed eight RBPs (PTRF, FNDC3B, SLC25A43, ZC3H12A, LRRFIP1, HSP90B1, HSPA5, and BNC2) are significantly associated with the survival of glioblastoma patients. Another 693 patients within the CGGA database were used to validate the findings. Additionally, 3564 RBPs were classified into canonical and non-canonical RBPs depending on the domains that they contain, and non-canonical RBPs account for the majority (72.95%). The Gene Ontology (GO) and Kyoto Encyclopedia of Genes and Genomes (KEGG) pathway analysis showed that some non-canonical RBPs may have functions in glioma. Finally, we found that the knockdown of non-canonical RBPs, PTRF, or FNDC3B can alone significantly inhibit the proliferation of LN229 and U251 cells. Simultaneously, RNA Immunoprecipitation (RIP) analysis indicated that PTRF may regulate cell growth and death- related pathways to maintain tumor cell growth. In conclusion, our findings presented an integrated view to assess the potential death risks of glioblastoma at a molecular level, based on the expression of RBPs. More importantly, we identified non-canonical RNA-binding proteins PTRF and FNDC3B, showing them to be potential prognostic biomarkers for glioblastoma.

## 1. Introduction

RNA-binding proteins (RBPs) are inherently pleiotropic proteins, regulating gene expression at the post-transcriptional level by interacting with target RNAs [1,2,3]. Previous studies have shown that RNA-binding proteins are involved in RNA metabolism [4] and play vital roles in regulating RNA stability, alternative splicing, modification, location, and translation [5]. Altering the expression of intracellular RBPs could influence a variety of physiological processes in cells, such as alternative splicing and apoptosis [6,7]. RNA-binding domains (RBDs) are the regions of RBPs that have been experimentally or structurally confirmed to bind RNAs directly [1]. Proteins containing RBDs are called classical or canonical RBPs. However, some RNA-binding proteins have not been proved to have the established domains by direct experimental evidence. Here, we temporarily called them non-classical or non-canonical RBPs. Over the past few decades, it has been reported that some metabolic enzymes also have RNA-binding abilities and can regulate the expression of their target RNAs [8,9,10,11], such as aconitase 1(ACO1), glyceraldehyde-3-phosphate dehydrogenase (GAPDH), and inosine 5′-monophosphate dehydrogenase 1 (IMPDH1) [8], which were named “moonlighting RBPs”. However, most of the moonlighting RBPs still need to be validated in living cells or animal models. Moreover, a recent research found that RBPs can interact directly with chromatin to regulate gene expression at the epigenetic level [12]. Studies in genetics and proteomics have shown that most RBPs exhibit functional abnormalities in many human diseases, including the human fragile X syndrome, myotonic dystrophy, nervous system diseases, and cancer [13,14,15,16].

Glioma is a kind of intracranial malignant tumor derived from neuroepithelium. According to the malignant degree of glioma, the World Health Organization (WHO) classified glioma into grades I–IV [17]. With the most aggressiveness and highest mortality rate, grade IV glioma is also referred to as glioblastoma (GBM), which represents 15% of central nervous system tumors [18]. The median survival of GBMs is less than 15 months [19]. Studies demonstrated that the expression levels of some RBPs are closely correlated with the malignancy of glioma. For instance, polypyrimidine-tract binding protein (PTBP1 or PTB), an alternative splicing factor, exhibits a higher expression level in most GBMs [20]. In addition, the splicing regulator, SNRPB, is also upregulated in gliomas, which has been identified as an oncogenic candidate RBP [21]. On the contrary, RNA-binding protein LARP4B serves as a tumor suppressor, with lower expression levels in gliomas [22]. Moreover, previously, we revealed that PCBP2 is overexpressed in gliomas [23], and clinical studies also demonstrated that GBMs with a high expression of PCBP2 have a poor prognosis [24]. In addition to participating in the initiation and progression of glioma, RBPs may also play an important role in the treatment of glioma. The RNA-binding protein, cold shock domain-containing protein E1 (CSDE1, also known as N-ras upstream gene protein, UNR), could function as a target of the anti-glioma stem cell drug, clofoctol, for tumor growth [25]. However, most of the RBPs that participate in glioma tumorigenesis and progression have remained relatively unexplored.

Currently, the diagnostic methods of glioma mainly depend on histopathological examination, imaging tests, and molecular diagnostics, such as IDH mutation and 1p/19q codeletion [26]. Therapies of glioma generally adopt traditional treatments, such as radiotherapy, chemotherapy, and surgical resection. However, due to the high heterogeneity and complexity of gliomas, these methods and treatments have little effect on the survival of patients. However, no substantial progress has been made in improving the prognosis of GBM. To further improve the diagnosis of glioma and ameliorate the quality of life and survival time of patients, finding new efficient molecular markers for diagnosis, treatment, and prognosis is an urgent matter. Therefore, a systematic functional study of RBPs will not only help to understand the pathogenesis of glioma, but also contribute to discovering new diagnostic or prognostic markers and screening innovative drug targets.

In this study, we utilized the weighted gene co-expression Network Analysis (WGCNA) to construct the link between the expression of RBPs and GBM patients’ clinical features. As a method of bioinformatics research, WGCNA is widely used to reveal the relevance of genes for different samples in order to find candidate biomarkers [27,28]. GO and KEGG pathway enrichment analyses were performed to reveal the underlying functional mechanisms of RBPs in gliomas. Moreover, we identified a cluster of RBPs involved in GBM progression, some of which could be used as potential prognostic or diagnostic biomarkers in the future.

## 2. Results

### 2.1. RNA-Binding Proteins Show a Significantly Higher Expression Than Transcription Factors in Glioma

**Figure 1:**
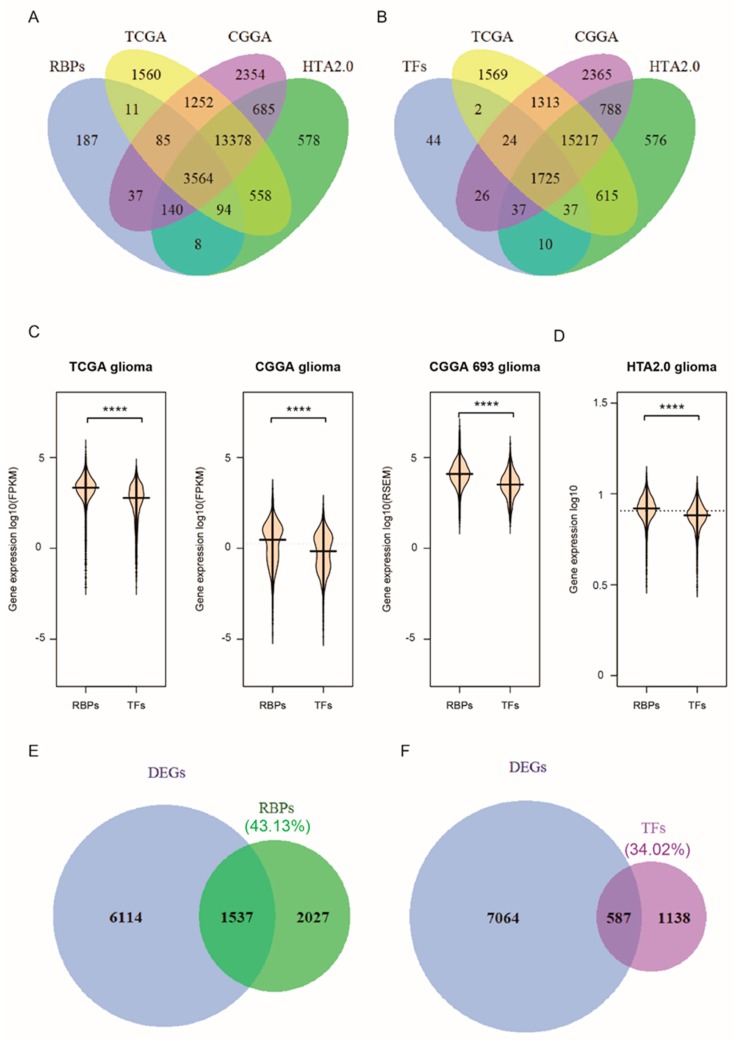
RNA-binding proteins show a significantly higher expression than transcription factors in glioma. (**A**) Venn diagrams of RNA-binding proteins in human glioma, (**B**) The transcription factors (TFs) in three glioma datasets, (**C**) bean plot of RBPs’ and TFs’ normalized expression in TCGA (left), CGGA datasets (middle) and the new CGGA dataset (right), (**D**) bean plot of RBPs’ and TFs’ normalized expression in our Microarray dataset, HTA2.0, which includes three glioma samples. The *p*-values were calculated using a Wilcoxon test by R (version 3.4.1) (*p* < 0.0001). (**E**,**F**) Venn diagrams of differentially expressed RBPs (**E**) and TFs (**F**). Differentially expressed genes (DEGs) analysis of GBM in the TCGA dataset was conducted by the R package, DEseq2 (*p* < 0.05). (**** *p* < 0.0001).

RBPs regulate gene expression at the post-transcriptional level by interacting with target RNAs. Relatively, the transcription factors (TFs) regulate gene expression mainly at the transcriptional level. We obtained data from Gerstberger [1], SONAR [29], the Gene Ontology project, Poly(A) binding protein [30,31,32,33,34], CARIC [35], and XRNAX [36], reported in the literature, reaching a total of 4127 RBPs in humans (Figure S1A). Previous research reported that there are ~20,352 potential protein-coding genes in the human genome [37], and only 7.5% are RBPs, while here we determined that RBPs account for ~20.28% (Figure S1B) of human protein-coding genes, which is ~2-fold increase. However, the 1905 TFs obtained from the literature reports [1,38] account for only ~9.36% (Figure S1B). This implies that RBPs may have a broader functional role than TFs. The deregulation of RBPs’ expression can be seen in a variety of diseases, including cancers. In this study, we examined the expression of 3564 RBPs (Figure 1A) and 1725 TFs (Figure 1B) in three different databases: RNA-seq data for 699 glioma samples and five normal samples from TCGA, RNA-seq data for 325 glioma samples from CGGA, and HTA2.0 chip data, consisting of three glioma samples and three normal samples. Consistent with previous studies, the abundance of RBPs (FPKM or RSEM) is significantly higher than that of TFs (*p* < 0.0001, Wilcoxon test) (Figure 1C,D). A total of 7651 differentially expressed genes (DEGs) (Figure S1C), comprising 1537 RBPs and 587 TFs, were obtained after the analysis of the TCGA datasets (Figure 1E,F, Table S1). Of the 3564 RBPs, the differentially expressed RBPs accounted for ~43.13%. In contrast, among the 1725 TFs, the differentially expressed TFs accounted for only 34.02%. Therefore, RBPs may play a more important role in glioma.

### 2.2. Associations between RBPs Expression and Molecular or Clinical Features in Glioma Patients

**Figure 2:**
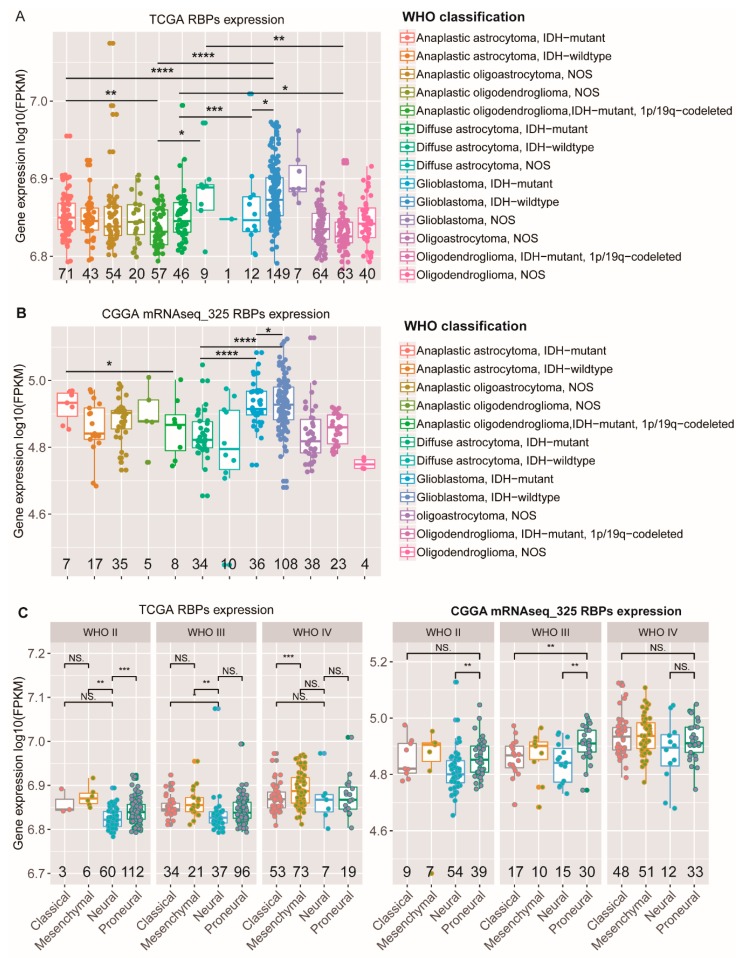
Associations between the expression of RBPs and molecular or clinical features in glioma patients. (**A**,**B**) RBPs’ normalized expression of different WHO classification gliomas in the TCGA (**A**) and CGGA (**B**) datasets. (**C**) RBPs normalized expression, stratified by the molecular subtype in TCGA glioma patients (left) and CGGA gliomas (right). (The *p*-values were calculated using a Wilcoxon test by R (version 3.4.1), * *p* < 0.05, ** *p* < 0.01, *** *p* < 0.001, **** *p* < 0.0001, NS: No significance, the numbers under all the graphs show the amount of samples.).

Next, we analyzed the expression patterns of RBPs in different types of glioma patients and compared their abundance in expression levels. In our studies, a global analysis of the expression of the mRNA levels of RBPs revealed that the abundance of RBPs increases with the grade (Figure S2A). Glioma patients were reclassified into 14 types in TCGA (Figure 2A), and patients from the CGGA database (mRNAseq_325) were reclassified into 12 types (Figure 2B), based on the 2016 WHO classification of central nervous system tumors [17]. Here, we analyzed the expression levels of RBPs based on RNA sequencing data on patients with different tumor types. The results showed that the expression of RBPs in GBMs with IDH mutation was relatively lower than that of the wildtype (Figure 2A,B). Simultaneously, we noticed that double-positive (IDH mutation plus 1p/19q codeletion) anaplastic oligodendroglioma patients showed significantly a lower RBP abundance compared with single-positive (IDH mutation only) anaplastic astrocytoma patients (Figure 2A,B). Then, we further explored the expression of RBPs in different molecular subtypes of gliomas in the TCGA and CGGA datasets. Compared with classical, mesenchymal, and pro-neural gliomas, RBPs were significantly downregulated in the neural subtype, independent of the grade (Figure 2C). In addition, we also assessed the relationship between the expression of RBPs and the MGMT (O6-methylguanine–DNA methyltransferase) promoter status. The promoter methylation of the MGMT gene has been proved to be more sensitive to temozolomide and associated with a longer overall survival of patients [39]. However, we did not find differences in the abundance of RBPs between MGMT methylated and unmethylated GBM patients (Figure S2B). In low-grade gliomas (LGGs), there are statistical differences, and these changes in the TCGA and CGGA datasets are not consistent. In TCGA, the results showed that the expression of RBPs in LGGs with MGMT methylation was relatively lower than that of the wildtype; however, the expression of RBPs in LGGs with MGMT methylation was relatively higher than that of the wildtype in CGGA (Figure S2B). The explanation for this phenomenon needs further investigation. A variety of molecular alterations occur in the process of glioma formation [40], which are closely linked to the clinical phenotype of glioma and can be used to predict the response to therapy and outcome of gliomas. To determine the relevance of RBPs to gliomas with different molecular alterations, we selected samples with TERT mutation (TCGA 166, CGGA 94) in the TCGA and CGGA datasets. In gliomas with TERT mutation, there is no significant difference in the abundance of RBPs, compared to the wildtype (Figure S2C, *p* > 0.05). Additionally, we found that the expression of RBPs in GBMs with TERT mutation was significantly higher than that of oligodendrogliomas with TERT mutation (Figure S2D).

### 2.3. Functional Enrichment Analysis of Canonical and Noncanonical RBPs Based on RNA-Binding Domains

**Figure 3:**
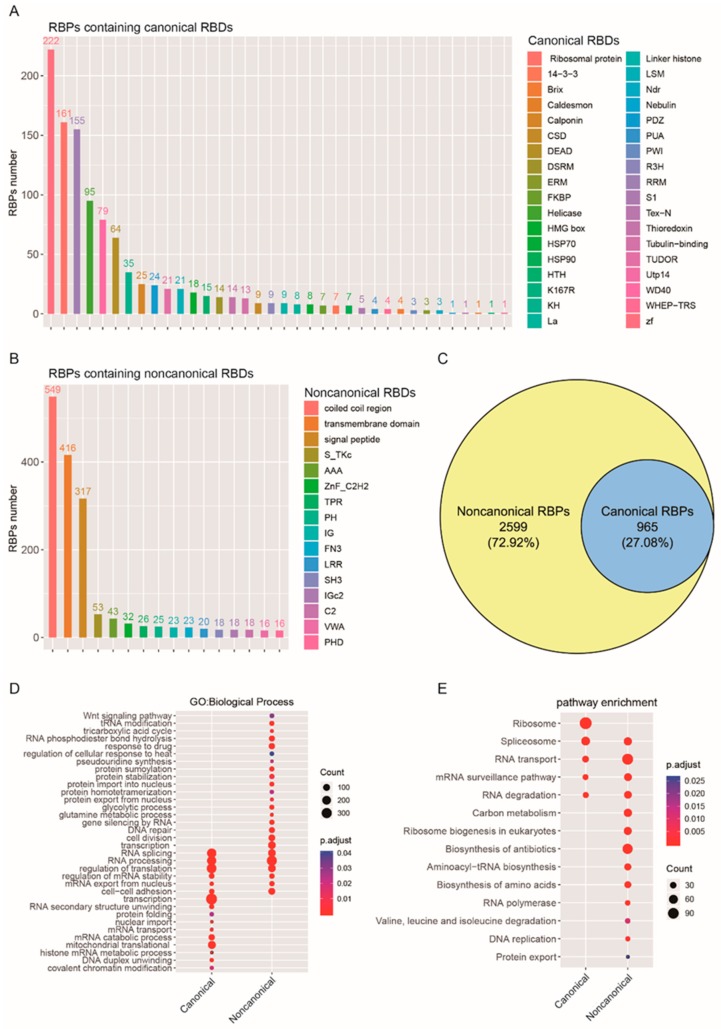
Functional enrichment analysis of canonical and noncanonical RBPs based on RNA-binding domains. (**A**,**B**) Canonical RBDs (**A**) and Noncanonical RBDs (**B**) of RBPs in human glioma. (**C**) The noncanonical RBPs constitute the majority of the RBPs in gliomas, (**D**) GO enrichment analysis of canonical and noncanonical RBPs based on biological processes. The *y*-axis shows a significantly enriched project (*p* < 0.05, *FDR* < 0.05). (**E**) KEGG pathway enrichment analysis of RBPs revealed that the noncanonical RBPs were enriched in the metabolism-related pathway. The *y*-axis shows a significantly enriched project (*p* < 0.05, *FDR* < 0.05).

Candidate canonical RBDs mainly include the RNA recognition motif (RRM), zinc fingers (ZF), tryptophan-aspartic acid 40 (WD40), K homology (KH) domain, double-stranded RNA-binding motif (DSRM), TUDOR domain, cold shock domain (CSD), etc. (Figure 3A, see also Table S3). In non-canonical RBPs, 549 RBPs contain coiled coil regions, and 416 RBPs contain transmembrane domains (Figure 3B, Table S3), but most of the non-canonical RBPs contain specific functional domains, and there is no direct experimental evidence proving that they have an RNA-binding function. Based on this, we reclassified 3564 RBPs related to glioma (Figure 1A), according to their different domains. For the 3564 RBPs in glioma, 2599 of them contain non-canonical RBDs. Among the RBPs, only 965 of them contain canonical RBDs (Figure 3C). Then, we annotated canonical RBPs’ and non-canonical RBPs’ functions using gene ontology (GO) pathway analysis for biological processes (Figure 3D, Table S3). The results revealed that canonical RBPs are enriched in transcription, translation, RNA splicing, and processing-related biological processes. Furthermore, mitochondrial translation-related biological processes are also significantly enriched in canonical RBPs (Figure 3D, *p* < 0.05, *FDR* < 0.05). In the non-canonical RBPs’ enrichment analysis, some biological processes were found to overlap with the canonical RBPs enrichment results. At the same time, some biological processes are specifically enriched in protein modification, stabilization, localization, and metabolism (glutamine metabolic, glycolytic, and tricarboxylic acid cycle) (Figure 3D, *p* < 0.05, *FDR* < 0.05). Furthermore, the Kyoto Encyclopedia of Genes and Genomes (KEGG) pathway analysis also demonstrated that canonical RBPs are enriched in ribosome, spliceosome, RNA transport and degradation, and mRNA surveillance pathways. However, non-canonical RBPs were found to be enriched in a large number of metabolic pathways, including carbon metabolism, the biosynthesis of antibiotics, and the biosynthesis and degradation of amino acids (Figure 3E). In addition, we found that some non-canonical RBPs possess enzymatic functions, such as human serine hydroxy methyltransferase (SHMT1 and SHMT2) and malate dehydrogenase 1(MDH1) (Figure S1C, Tables S1 and S3). These RBPs are indispensable for carbon metabolism, which sustains growth and proliferation in normal and tumor cells.

### 2.4. RBPs’ Co-Expression Network Modules Identified by WGCNAs

**Figure 4:**
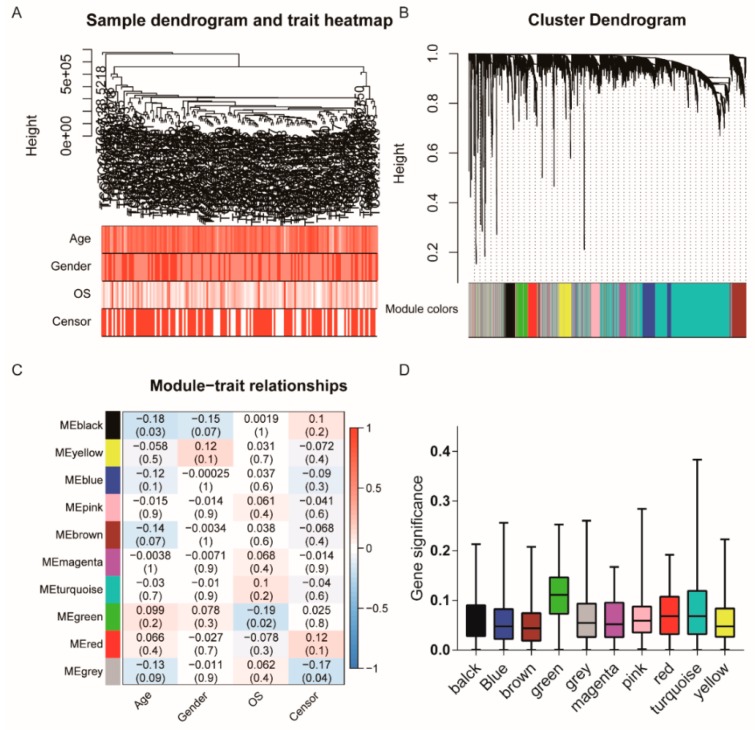
Co-expression Network modules of RBPs identified by WGCNAs and survival analysis. (**A**) cluster tree of human GBM samples in TCGA. The color band underneath the tree indicates the numeric values of the tissue traits, (**B**) Cluster Dendrogram indicating different RNA-binding protein modules. (**C**) The heat map visualizes the correlation between the modules and the patient’s clinical characteristics. The values indicate the correlation and *p*-value in squares. A positive score indicates a positive correlation. (**D**) The distribution of the average gene significance in different modules is shown.

To further identify the association between RBPs and gliomas with different clinical characteristics, the expression data profile of these 3564 RBPs from TCGA were transformed into a gene co-expression network using a WGCNA package in R. The analysis was performed as described previously [27]. First, we selected 160 patients from the TCGA glioblastoma dataset with different ages, genders, and overall survival (OS) rates for RBPs expression clustering (Figure 4A). A total of 10 co-expressed modules were obtained through a one-step network construction method, where β = 6 (Figure 4B, Figure S3A). Then, we found a strong similarity of five co-expression modules (Figure S3C). Furthermore, we performed a correlation analysis between different co-expression modules and glioma sample traits (Figure 4C). The results indicate that the black module has the most significant correlation with age (correlation coefficient = −0.18, *p* = 0.03). The green module was significantly correlated with the overall survival of patients (correlation coefficient = −0.19, *p* = 0.02, Figure 4C). However, modules that are significantly related to gender have not been found. The distribution of the modules’ average gene significance related to OS are shown in Figure 4D and Figure S3D, among which the green module was also found to have the strongest association with the overall survival (OS) of GBM patients. Thus, this module was selected as the clinically most significant module of prognosis for further analysis.

### 2.5. Survival Analysis of RBPs in the Green Module

**Figure 5:**
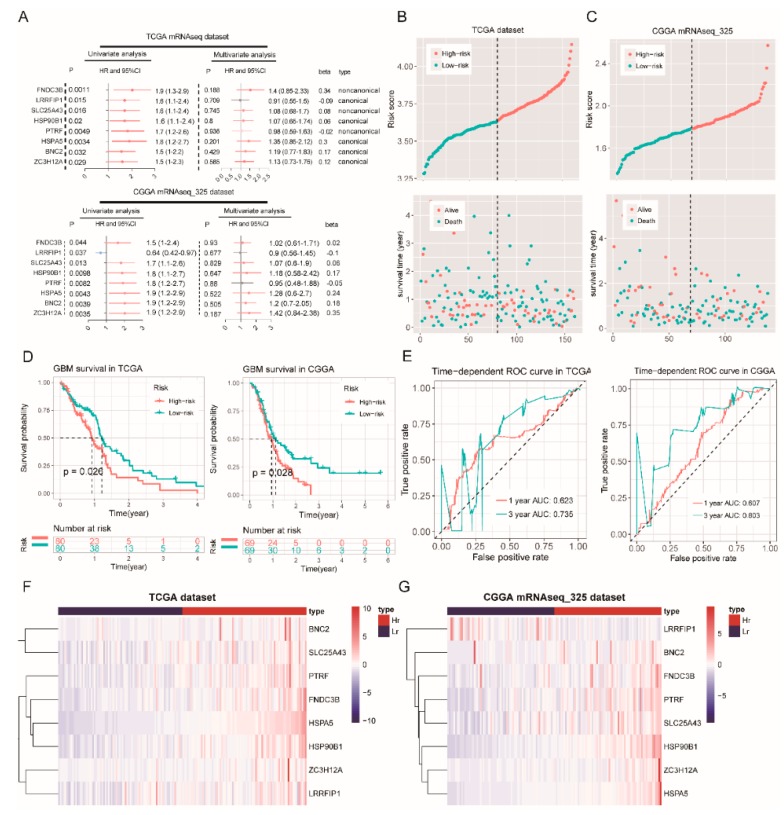
Survival analysis in the TCGA and CGGA mRNAseq_325 datasets. (**A**) the green module univariate and multivariate analysis in the TCGA (top) and CGGA mRNAseq_325 datasets (bottom), (**B**) the risk score distribution (top) and survival status distribution (bottom) for 160 GBM patients (TCGA dataset), (**C**) The risk score distribution (left) and survival status distribution (right) for 138 GBM patients (CGGA mRNAseq_325 dataset), (**D**) the Kaplan–Meier survival curves for the high- and low-risk groups in the TCGA (left) and CCGA mRNAseq_325 datasets (right), (**E**) the ROC curves for GBM patients, predicting the OS by the risk score in the TCGA (left) and CCGA mRNAseq_325 datasets (right), (**F**,**G**) a heat map of eight RBPs for the high- and low-risk groups (Hr: High-risk; Lr: Low-risk).

We identified eight prognosis-related RBPs in the green module by Cox regression analysis (Figure 5A). These eight RBP risk ratios (HR) are greater than one (Figure 5A). A prognostic gene signature composed of eight RBPs was developed after using the multivariate Cox proportional hazards regression model. A total of 80 patients with risk scores larger than the median risk score (3.65) were placed in the high-risk group, whereas the other 80 patients were placed in the low-risk group in the TCGA dataset (Figure 5B). Moreover, a total of 69 patients with risk scores larger than the median risk score (1.79) were placed in the high-risk group, whereas the other 69 patients were placed in the low-risk group in the CGGA mRNAseq_325 dataset (Figure 5C). The results of survival analysis based on risk scores indicate that patients with high risk scores have a shorter survival time (Figure 5D). Finally, the ROC curve presented a relatively good performance in survival prediction, as the AUC was 0.623 (TCGA, 1-year OS), 0.735 (TCGA, 3-year OS), 0.607 (CGGA, 1-year OS), and 0.803 (CGGA, 3-year OS) (Figure 5E). However, these RBP expression levels of mRNA are increased in the high-risk groups (Figure 5F,G), which suggests that a high expression of these RBPs is associated with a poor prognosis of GBM.

To further verify the reliability of the results, we downloaded another glioblastoma dataset (dataset ID: mRNAseq_693) in the CGGA database. This dataset includes 205 GBM patients. Similar results to the results of survival analysis in this dataset were obtained (Figure S4A–D).

### 2.6. Non-Canonical RBPs Were Involved in Maintaining Cell Growth

**Figure 6:**
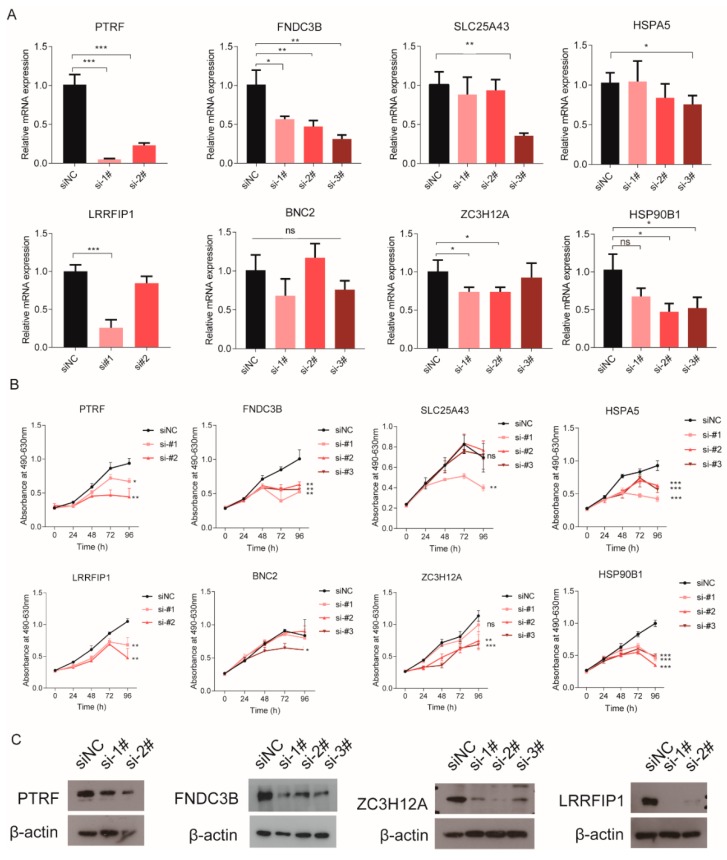
An MTS assay was used to measure the proliferation of the LN229 cell. (**A**) real-time quantitative PCR for the expression of eight RBPs in different groups, (**B**) proliferation of the LN229 cell after knocking down eight RBPs, (**C**) Western blot used to detect the knockdown effect of PTRF, FNDC3B, ZC3H12A, and LRRFIP1 (siNC: Negative Control; si-1#, si-2# and si-3#: They are different knockdown sequences of target genes, * *p* < 0.05, ** *p* < 0.01, *** *p* < 0.001).

We have shown that eight poor prognosis-related RBPs include three non-canonical RBPs and five canonical RBPs (Table 1 and Figure 5A). To investigate whether these RBPs have biological functions, we designed some siRNAs (see Materials and Methods) to decrease the expression of these RBPs in glioma cells. Real-time PCR showed that the expression of these RBPs was decreased in the LN229 cell line (Figure 6A). Of the three non-canonical RBPs, PTRF or FNDC3B knockdown significantly inhibits the proliferation of the GBM cell line, LN229 (Figure 6B). Among the five canonical RBPs, LRRFIP1, ZC3H12A, and HSP90B1 silencing suppresses the proliferation of the LN229 cell (Figure 6B). No obvious biological effects were observed for BNC2 depletion in the LN229 cell (Figure 6B). The protein expression of PTRF, FNDC3B, ZC3H12A, and LRRFIP1 was also validated by Western blot (Figure 6C and Figure S6A). Among them, we paid special attention to the non-canonical RNA-binding proteins.

Functional study of non-canonical RBPs in gliomas is a new research direction. In our research, we found that PTRF, which is a novel non-canonical RNA-binding protein, participates in maintaining GBM proliferation. To further confirm these results, PTRF siRNAs were used to knock down PTRF in another glioma cell line, U251 (Figures S5A and S6B). PTRF depletion also suppressed cell proliferation in U251 (Figure S5B). A similar result was also obtained after we knocked down FNDC3B in U251 (Figure S5). All the results described above indicated that the two non-canonical RBPs have biological functions in glioma, and they can be used as the potential prognostic biomarkers for GBM.

### 2.7. RIP Assay Analysis of the Targets of the Non-Canonical RNA Binding Protein PTRF

**Figure 7:**
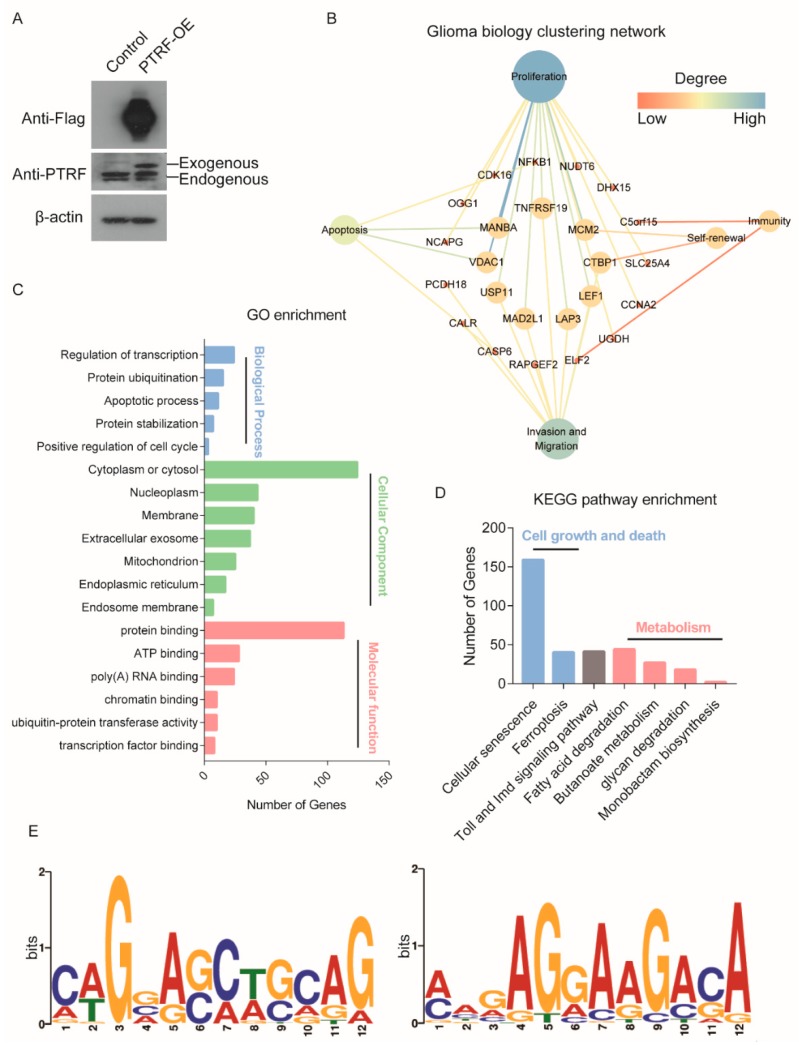
RIP-seq analysis. (**A**) Western blot confirming the expression of PTRF in the Flag-PTRF overexpression’s LN229 cell, (**B**) the glioma biology clustering network of target genes, (**C**) gene ontology enrichment analysis of the target genes of PTRF, (**D**) KEGG pathway analysis of the target genes of PTRF, (**E**) top two consensus motifs in the LN229 cell with Flag-PTRF overexpression.

To further study the potential mechanism of PTRF as an RNA-binding protein affecting glioma cell growth, we performed an RIP experiment in LN229 cells with PTRF overexpression (Figure 7A and Figure S6C). Then, target genes that were specifically bound by PTRF were identified by high-throughput sequencing. We also obtained glioma biology-related target RNAs, and most target RNAs are involved in glioma proliferation (Figure 7B). Moreover, the GO enrichment analysis was performed in the target genes of PTRF. These targets were mainly located on cytoplasm or cytosol (Figure 7C). The biological processes’ enrichment results showed that the significant enrichment included a regulation of transcription, protein ubiquitination, apoptotic process, and cell cycle (Figure 7C). To further identify target gene-related pathways, we performed KEGG pathway enrichment analysis. The results showed that the target genes were mainly enriched in cell senescence, cell ferroptosis, and cell metabolism pathways (Figure 7D), such as fatty acid degradation, glycan degradation, and butanoate metabolism. In order to detect the preference of the nucleic acid sequence of the PTRF protein-specific binding sites and the motif sequences information, the MEME Suite software (Version 5.1.1, university of Nevada, Reno, San Francisco, America) was used to perform motif analysis. The results showed that the motif sequences had relatively high cytosine and adenine conservation (Figure 7E).

## 3. Discussion

Glioma is a kind of brain cancer, with the highest mortality rate among such cancers. There is no targeted therapy to ensure the greatest survival rate for glioma patients. All the therapies that have demonstrated a significant survival rate for gliomas use nonspecific targeting of cancer cells, such as radiation, chemotherapy (temozolomide), and surgery [41]. In recent years, in order to improve the treatment effect of glioma, some glioma subtypes have been identified based on molecular genetic features [42], such as IDH mutation [43], TERT promoter, and 1p/19q codeletion. These molecular markers mainly relied on the mutation. However, RBPs are always independent from mutations [14], and their expression changes can cause malignant progression in glioma. Studies show that RBPs are dysregulated in many cancer types, including glioma [44].

In this study, the abundance of RBPs is significantly higher than that of TFs, which is consistent with previous studies [45]. Meanwhile, comparison of the numbers of differentially expressed RBPs and TFs further illustrates the key role of RBPs in the development of glioma. Additionally, we found that canonical and non-canonical RBPs could regulate glioma progression through different biological processes and pathways. The biological processes or pathways that canonical RBPs are mainly involved in include RNA splicing, processing, localization, transport, and stability. A small proportion of RBPs involved in RNA alternative splicing have been reported in glioma. For instance, PTBP1 can promote the proliferation and migration of glioma by enhancing the RTN4 mRNA inclusion of exon 3 [20]. In addition, PTB, hnRNPA1, and hnRNPA2 controlled by c-Myc regulate the mRNA splicing of pyruvate kinase, which is correlated with PKM2 expression [46]. The RNA-binding protein, HNRNPA2B1, can regulate the splicing of many tumor suppressor genes in glioma, such as RON, BIN1, WWOX, and c-FLIP. By enhancing the expression of the oncogenic isoforms of these genes, HNRNPA2B1 promotes glioma progression and aggressiveness [47]. However, non-canonical RBPs also play key roles in regulating tumor progression. In our analysis, we found that non-canonical RBPs can modulate the progression of tumors through some specific metabolism pathways, such as carbon metabolism and amino acid metabolism. A recent study identified that SHMT1 in the cytosol has RNA-binding activity. By binding to the 5′ untranslated region of the SHMT2 transcript (UTR2), SHMT1 regulates the expression of its mitochondrial counterpart (SHMT2) [48,49]. Furthermore, we noticed that both SHMT1 and SHMT2 are overexpressed at the mRNA level in GBM according to TCGA RNA sequencing data, which implies that SHMT might also play an important role in the development of GBM. MDH1 (malate dehydrogenase 1), a pleiotropic protein, was identified as a poly (A) RNA-binding protein in the Hela cell. In pancreatic ductal adenocarcinoma (PDAC), the knockdown of MDH1 or inhibition of its activity could repress mitochondria respiration and influence glutamine metabolism, which enhances tumor cells’ sensitivity to oxidative stress and suppresses proliferation [50]. Moreover, as a novel regulator of autophagy, MDH1 is required for autophagosome formation in the early stages of autophagy to maintain pancreatic tumor cell survival [51]. Here, we found that MDH1 is downregulated at the mRNA level in GBM based on the TCGA RNA sequencing data. However, there is no direct experimental evidence showing that MDH1 works as a new RBP participating in glioma progression. Altogether, canonical RBPs and non-canonical RBPs like metabolic-related enzymes could play key roles in the development of tumors.

According to the 2016 WHO classification of central nervous system tumors, glioblastoma was categorized into three types, including IDH mutation, IDH wildtype, and NOS [17]. The patients with mutant IDH GBM have a better prognosis than patients with wildtype GBM. However, the expression of RBPs is lower in GBM patients with IDH mutation in our results. This indicates that a high expression of the RNA-binding protein is associated with a poor prognosis. Then, the green module, identified by WGCNA, is significantly associated with the OS of GBM patients. The black module is significantly negatively correlated with the age of pathogenesis. By monitoring the expression of these RBPs, it is possible to reduce the risk of death of patients to a certain extent depending on the specific age. Survival analysis identified eight RBPs in the green module, which are significantly associated with a poor prognosis of glioma. Polymerase I and transcript release factor (PTRF), also known as Cavin1, has previously been described as a critical biomarker in both glioma and serum exosomes [52]. In GBM, the expression of PTRF is regulated by the EGFRvIII overexpression, which is related to the EGFR/PI3K/AKT pathway. Here, PTRF serves as a non-canonical RNA-binding protein and was also identified as a prognosis related factor in glioma. Fibronectin type III domain-containing protein 3B (FNDC3B) is a membrane protein. It was originally identified as a positive regulator of adipogenesis [53]. In acute promyelocytic leukemia (APL), FNDC3B may promote pathogenesis by fusing to retinoic acid receptor α (RARA) [54]. FNDC3B can also promote tumor migration and invasion. In our study, we found that FNDC3B not only acts as a prognostic biomarker, but can also promote glioma cell proliferation. Another non-canonical RBP solute carrier family 25 member 43 (SLC25A43) is a mitochondrial membrane-specific transporter, which is widely expressed in the central nervous system [55]. Moreover, in GBM, it was identified as a molecular marker with a poor prognosis. In this study, we also found five canonical RBPs associated with GBM prognosis. Finally, we identified the target mRNAs of PTRF in GBM and its potential functional pathways. However, the mechanism of PTRF needs further study. In addition, inhibition of PTRF expression may be an effective way to treat GBM in the future. Altogether, these eight RBPs may have a prognostic value for GBM, and an in-depth study of the biological functions of these RBPs suggested that the non-canonical RNA-binding protein, PTRF, might be particularly important for GBM progression.

## 4. Materials and Methods

### 4.1. Datasets and Patient Information Acquisition

We integrated a list of human RBPs from six sources: Gerstberger, SONAR, the Gene Ontology project, Poly(A)-binding protein, CARIC, and XRNAX. The list of human transcription factors comes from two studies. The transcriptome data on 699 patients were obtained from the Cancer Genome Atlas database (TCGA) [56]. We used 160 cases of GBM, with clinical information for survival analysis in TCGA. Meanwhile, we also extracted 325 samples (dataset ID: mRNAseq_325) of RNA-seq data from the Chinese Glioma Genome Atlas (CGGA) database [57]. In addition, 138 GBM patients with survival and clinical information were used for survival analysis. Of the 138 GBM samples, 54 were characterized as recurrent and secondary glioblastoma (rGBM, sGBM). The HTA 2.0 dataset (see Table S2 for details) contains 3 normal tissues and 3 tumor tissues. Additionally, we downloaded the latest 693 samples (dataset ID: mRNAseq_693) released by the CGGA database, and 205 GBM patients’ clinical and survival information were used to validate our results. A total of 503 GBM samples were used for the analyses.

### 4.2. RBPs Domains, Gene Ontology, and Pathway Enrichment Analysis in Gliomas

We extracted ~2878 protein domains of RBPs from the database Pfam (http://pfam.xfam.org) [58]. Then, according to the domain information, RBPs were classified into two families: the canonical subfamily with canonical RBDs and the non-canonical subfamily. We conducted Gene Ontology and Kyoto Encyclopedia of Genes and Genomes (KEGG) pathway enrichment analysis using the Database for Annotation, Visualization, and Integrated Discovery (DAVID, version 6.8) [59]. A *p*-value < 0.05 and *FDR* < 0.05 were used as the cut-off criteria.

### 4.3. Weighted Gene Co-Expression Network Analysis

The expression profile of 3564 RBPs was obtained from the TCGA database. After validation, these data were used to construct a co-expression network using the WGCNA package in R (version 3.6.0). The WGCNA methodology analysis was performed as described previously [27]. First, a hierarchical clustering analysis of glioma samples with different clinical characteristics (age, gender, OS, and Censor) was performed, based on the expression of RBPs, to remove outlier samples. Then, the fit soft threshold power (β) was screened to ensure the construction of scale-free networks, based on the Pearson’s correlation coefficient between RBPs. In this study, β = 6 (Figure S3A,B, scale free *R*^2^ = 0.89) was selected to construct a scale-free network. In light of the TOM-based dissimilarity measure, with a min-Module size (gene group) of 30 for the RBPs cluster dendrogram, average linkage hierarchical clustering was conducted, and RBPs with similar expression modes were classified into the same modules by a one-step network construction and module detection. Next, we defined two parameters. One is the module eigengenes (MEs), which represents the first principal component-related module, and its value can be considered to represent all genes in the module. The other is gene significance (GS), which was defined as the correlation between genes and traits and used to quantify the association of individual genes with the traits that we are interested in. According to these two parameters, we identified modules that are significantly associated with the clinical traits.

### 4.4. Survival Analysis

Univariate Cox regression analysis was performed to assess the prognostic value of RBPs associated with survival time, and RBPs correlated with the overall survival time (*p* < 0.05) were selected to achieve a further gene signature selection and risk-based classification in the TCGA and CGGA datasets (dataset ID: mRNAseq_325). A risk signature was formulated according to the multivariate Cox proportional hazards regression analyses. The risk score was formulated as previously reported [60]. The risk score for each glioma patient was calculated using the following formula:

Risk score = betagene1× exprgene1+ betagene2×exprgene2+ betagenen × exprgene

A regression coefficient (beta) was derived from the multivariate Cox regression analysis, and low- and high-risk groups were divided using the median risk score. Expr_genen_ represents the expression of genes. The formula was used to calculate the risk score in the TCGA and CGGA datasets.

### 4.5. Cell Culture and Lentiviruses Infection

The human GBM cell lines LN229 and U251 were purchased from the American Type Culture Collection (ATCC) and cultured in DMEM (Dulbecco’s Modified Eagle’s Medium) with 1% sodium pyruvate and 10% FBS (fetal bovine serum). The Flag-PTRF LN229 cell line was derived by transfecting lentiviruses, after puromycin sectioning, for 7 days. The Flag-PTRF lentiviruses (28888-1) and NC lentiviruses (Con335) were purchased from Shanghai Genechem. All cell lines were maintained in a humidified 5% CO_2_ atmosphere at 37 °C.

### 4.6. siRNA Transfections and Cell Proliferation Assay

siRNAs were purchased from the Gene Pharma corporation. The siRNA and sequences used are presented in Table S4. The gene knockdown was achieved via transfecting siRNA using INTERFERin^®^ (PolyPlus PT-409-10), following the manufacturer’s protocol. Cells Proliferation was detected by the CellTiter 96^®^ AQueous Non-Radioactive Cell Proliferation Assay (Promega, Madison, Wisconsin, America).

### 4.7. Quantitative Real-Time PCR and Western Blot Analysis

The total RNA was isolated using TRIzol reagent (Invitrogen, Carlsbad, State of California, America). cDNA synthesis, following the instructions of HiScript II Q RT SuperMix for qPCR (Vazyme R212) was performed at 50 °C for 15 min and 85 °C for 5 s. qPCR was performed using a SYBR green-containing PCR kit (Takara, Bei Jing, China). The relative gene expression was analyzed by the Comparative Cq Method (ΔΔCq). All samples were run in triplicate in each experiment. The qPCR primers are shown in Table S4. Protein was isolated using a TNTE lysis buffer, with four protease inhibitors. Additionally, the procedure refers to a previous protocol. The primary antibodies were as follows: anti-PTRF (Abcam, dilution 1:1000, ab48824, Cambridge Science park, United Kingdom), anti-FNDC3B (Proteintech Group, dilution 1:1000, 22605-1-AP, Wu Han, China), anti-ZC3H12A (Sigma-Aldrich, dilution 1:1000, SAB3500391, Saint Louis, State of Missouri, America), anti-LRRFIP1 (Santa Cruz Biotechnology, dilution 1:200, sc-101168), anti-Flag antibody (Sigma, dilution 1:4000, F7425, Saint Louis, State of Missouri, America), and anti-β-actin(Sigma, dilution 1:5000, A5441, Saint Louis, State of Missouri, America) antibodies.

### 4.8. RNA Immunoprecipitation and High-Throughput Sequencing

RNA immunoprecipitation (RIP) analysis was performed according to the protocol provided by Medical and Biological Laboratories Co. Ltd. (MBL, Bei Jing, China) and Ribocluster Profiler RIP-Assay kit (catalog RN1001, MBL). Flag-PTRF overexpression cells were collected in 1.5 mL centrifuge tubes from the 100 mm culture dish using a cell scraper. We prepared 10 million cells per sample, and each sample had two biological replicates. Next, cells were washed twice with nuclease-free ice-cold PBS. The cell suspension was centrifuged at 300× *g* for 5 min at 4 °C. After removing the supernatant, 500 μL of lysis buffer(+ 1mM Dithiothreitol) was added to each sample, and the mixture was vortexed thoroughly. The sample was incubated for 10 min on ice and centrifuged at 12000× *g* for 10 min at 4 °C. The cell pellet was removed, and the supernatant was transferred to the tube containing Pierce™ Anti-DYKDDDDK Magnetic Agarose (Invitrogen, A36798, Carlsbad, State of California, America), and then washed once with the lysis buffer (+ 1 mM Dithiothreitol). The tube was incubated with rotation overnight at 4 °C. The tube was placed on a magnetic stand, and the supernatant was removed. Beads were used to isolate and purify the RNA. Purified RNA was used for reverse transcription and the library construction of high-throughput sequencing. RIP experiments were performed in two biological replicates.

### 4.9. Statistical Analysis

Here, we evaluated the expression levels of ~3,564 RBPs. After normalization, the sum value was used as the expression abundance of RBPs and TFs in a single patient. The DEGs were analyzed by the DEseq2 package in R (version 3.4.1) [61]. Boxplot generation and statistical analysis of the expression difference were conducted using the R (version 3.4.1) packages, “ggplot2”, “ggpubr” and “ggsignif”. The univariate and multivariate Cox proportional hazards regression analyses were performed utilizing a “survival” package in R (version 3.4.1). A survival curve made using the Kaplan–Meier method was implemented to estimate the differences in the overall survival between the low- and high-risk gliomas. All the statistical analyses were conducted using R (version 3.4.1) and SPSS.22.

## 5. Conclusions

Overall, we comprehensively analyzed the key RBPs modules specifically associated with the overall survival of glioma using bioinformatics analysis. Moreover, we identified some non-canonical “moonlighting” RBPs that are differentially expressed and associated with carbon metabolism in glioma. The survival analysis revealed that a cluster of RBPs might have a prognostic value for GBM. Moreover, further studies identified that non-canonical RNA-binding proteins PTRF and FNDC3B suppressed glioma cell growth and found that they can be used as potential prognostic biomarkers for GBM. Finally, RIP-seq analysis indicates that PTRF may regulate cell growth- and death-related pathway for maintaining tumor cell growth.

## Figures and Tables

**Table 1:** The source of eight prognosis-related RBPs in the green module.

RBP	SONAR	Gerstberger	GO: RNA Binding	Poly(A) Binding Protein	CARIC	XRNAX	Type
PTRF		√	√	√			Non-canonical
FNDC3B	√		√	√	√	√	Non-canonical
SLC25A43	√						Non-canonical
LRRFIP1		√	√			√	Canonical
ZC3H12A		√	√				Canonical
HSP90B1			√	√		√	Canonical
HSPA5				√		√	Canonical
BNC2	√						Canonical

## Supplementary Materials

The following are available online at https://www.mdpi.com/2072-6694/12/4/892/s1, Figure S1: The RNA-binding proteins in the human genome; Figure S2: Associations between the expression of RBPs and molecular or clinical features in glioma patients related to Figure 2; Figure S3: WGCNA analysis in GBM relative to Figure 4; Figure S4: Survival analysis verification of the prognostic gene signature of eight RBPs in GBM patients (CGGA mRNAseq_693 dataset) related to Figure 5; Figure S5: Detection and verification of the cell proliferation ability in U251 cells related to Figure 6. Figure S6: The uncropped western blot figures were shown with intensity ratio. Table S1: Differentially expressed genes in GBM, Table S2: HTA2.0 dataset, Table S3: RNA-bingding proteins domain enrichment analyses, Table S4: The sequences of primers and siRNAs.
