# Supplementary material for: Integrated Analysis of RNA-Binding Proteins in Glioma
Source: Cancers (Basel). 2020 Apr 7;12(4):892. doi: 10.3390/cancers12040892 (PMC7226056; doi:10.3390/cancers12040892)
Supplement: Supplementary file 1 [file cancers-12-00892-s001.zip › cancers-751631-supplementary materials/cancers-751631-suppl-final.docx]

Supplementary Materials: Integrated Analysis of RNA-Binding Proteins in Glioma

Zhixing Wang, Wanjun Tang, Jiangang Yuan, Boqin Qiang, Wei Han and Xiaozhong Peng


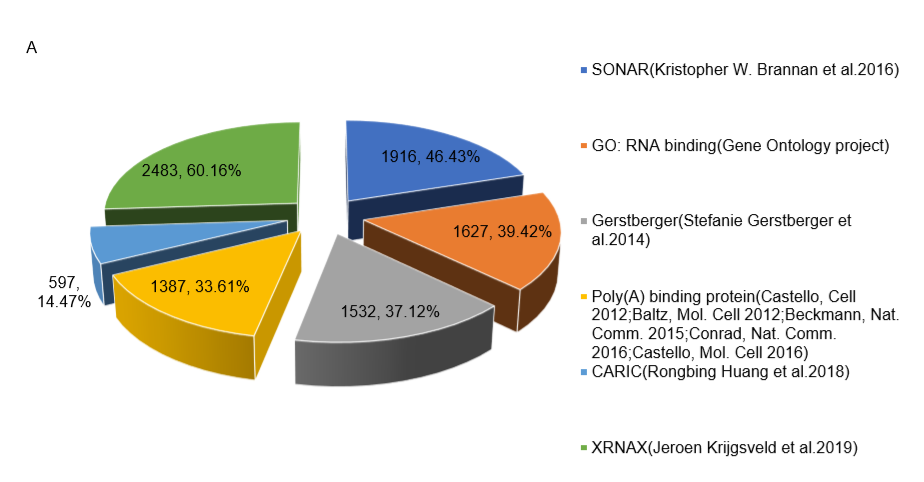


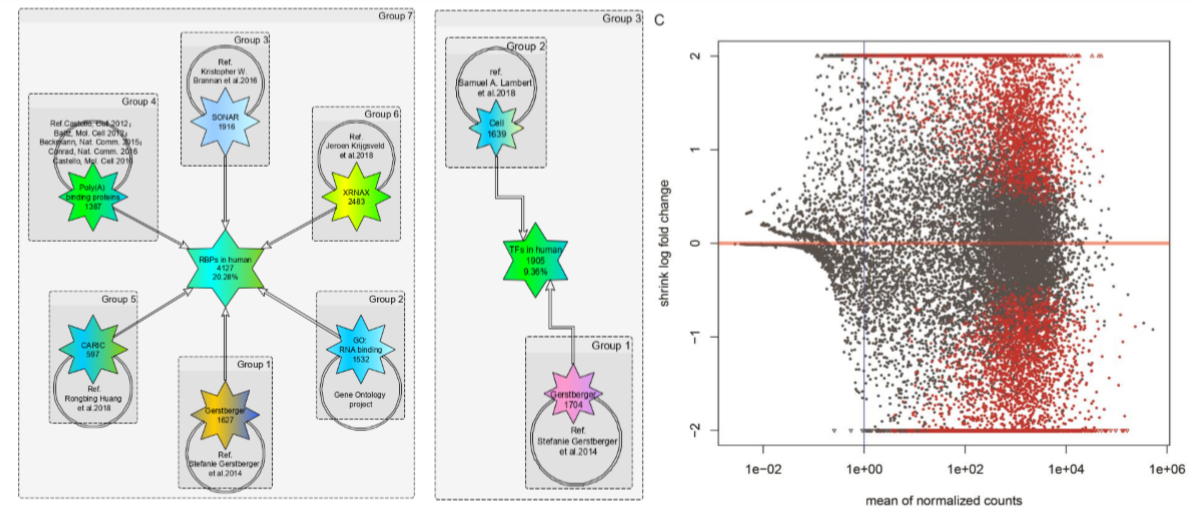


**Figure S1.** The RNA-binding proteins in human genome. (**A**) The human RNA-binding proteins were integrated by literature reports; (**B**) RBPs as a percentage of human genomic protein-encoding genes; (**C**) Distribution of differential expression genes in TCGA glioblastomas.


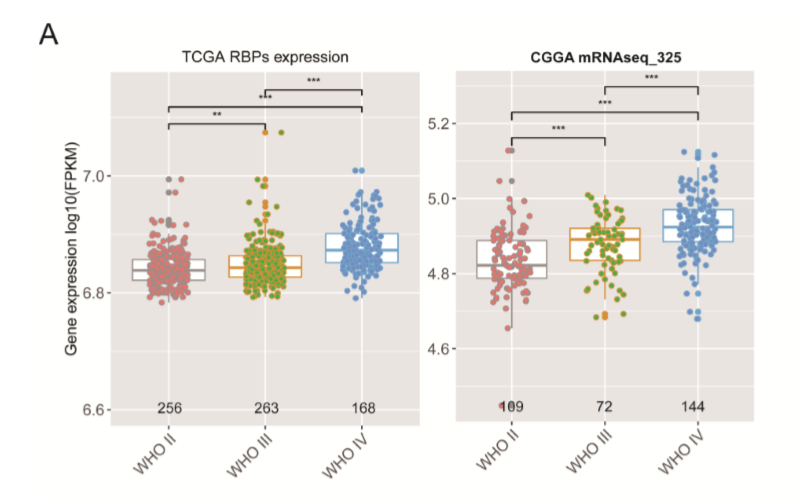


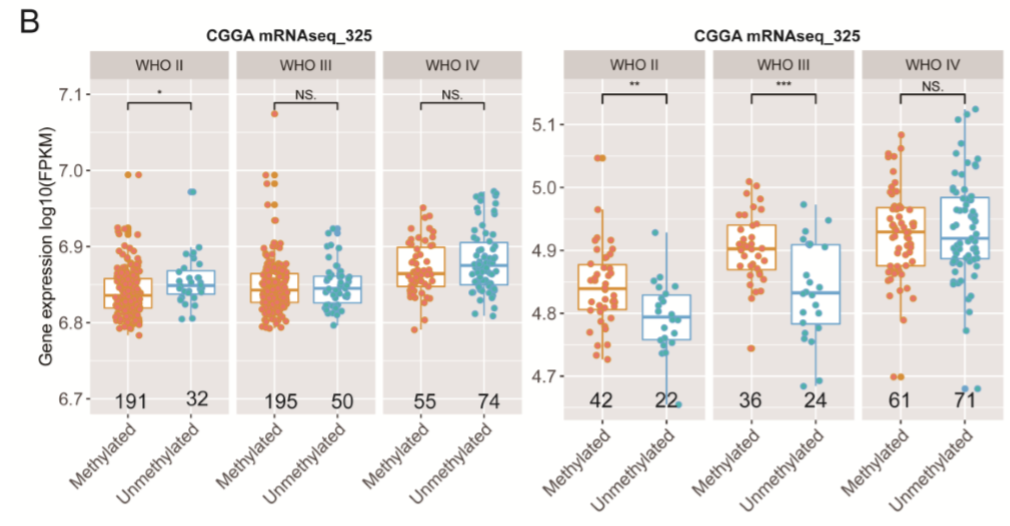


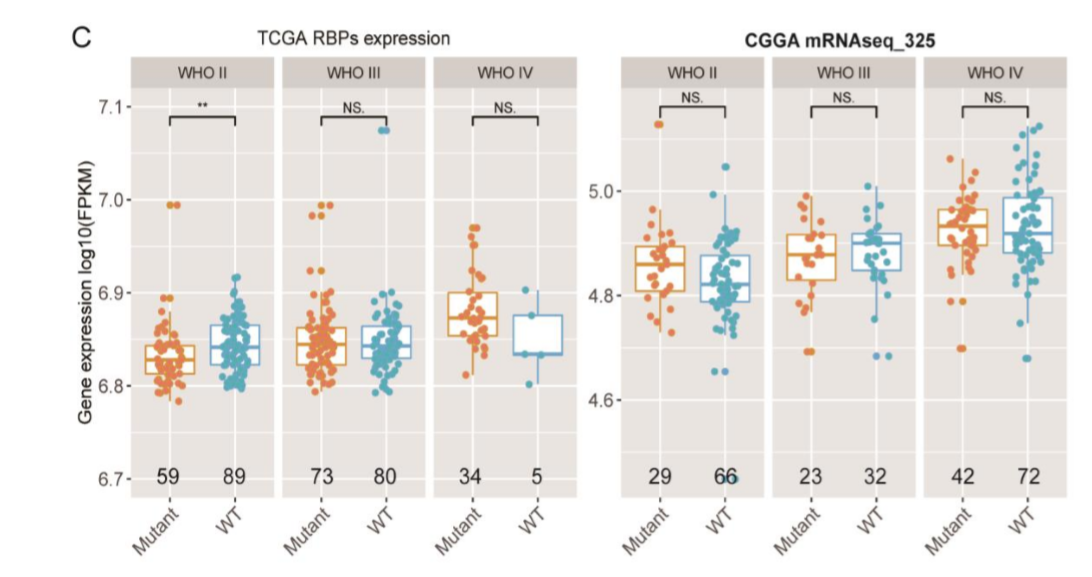


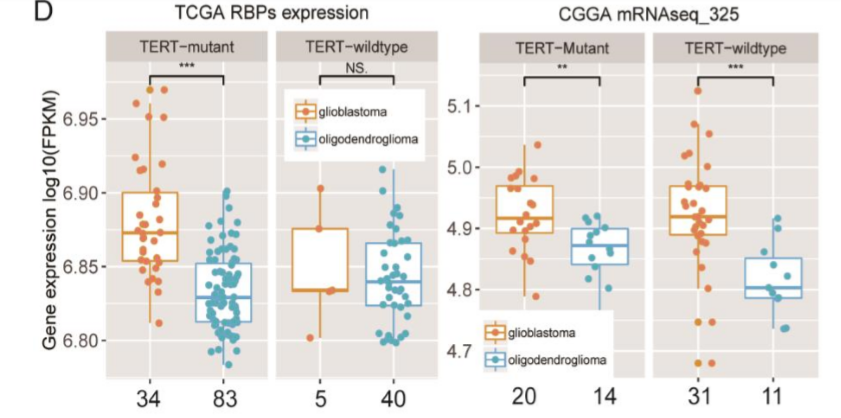


**Figure S2.** (**A**) RBPs’ normalized expression of different grade gliomas in TCGA (top) and CGGA (bottom) datasets; (**B**) The difference in expression of RBPs is based on the MGMT status; (**C**) RBPs normalized expression in different groups classified by TERT promoter status. TCGA glioma samples (left), CGGA glioma patients (right); (**D**) RBPs normalized expression in TERT mutant glioblastoma and oligodendroglioma. (The *p* values were calculated using a Wilcoxon test by R (version 3.4.1), * *p* < 0.05, ** *p* < 0.01, *** *p* < 0.001, NS: No significance, the numbers under all the graphs show the amounts of samples.).


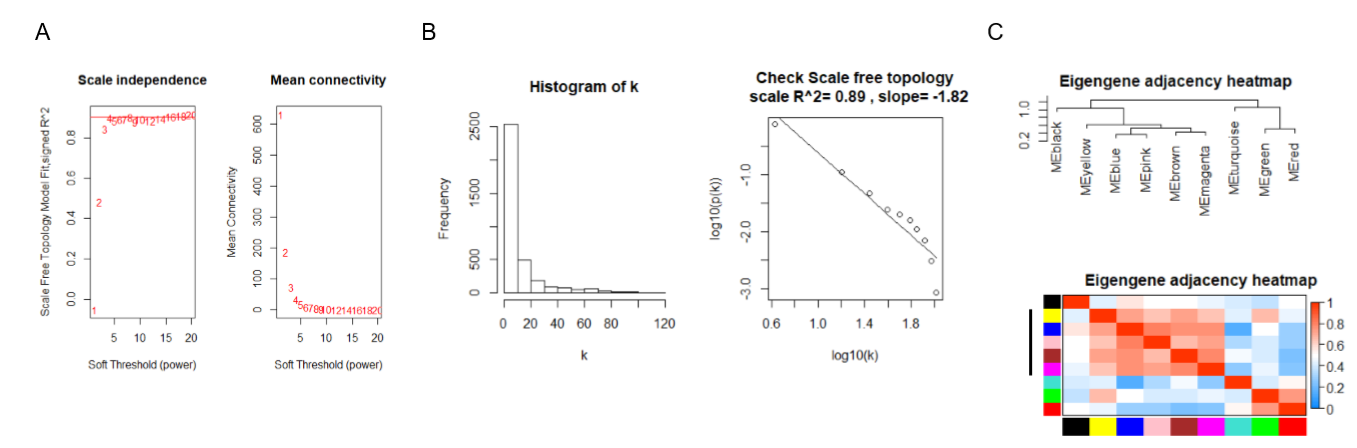


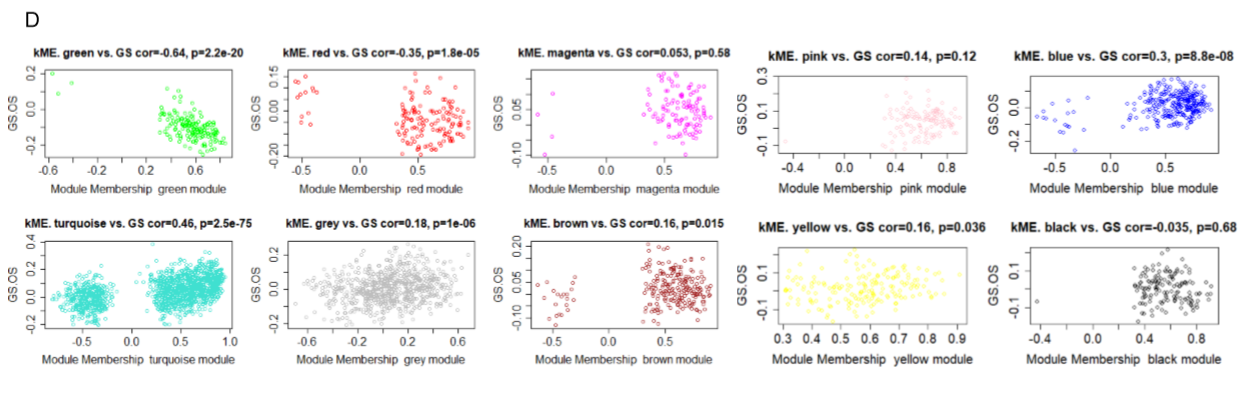


**Figure S3.** WGCNA analysis in GBM. (**A**) Analysis of the scale-free fit index for various soft-thresholding powers (left) and the mean connectivity for various soft-thresholding powers (right); (**B**) Checking the scale free topology when β = 6. (**C**) Visualization of the relationships between modules. (**D**) Correlation between RBP and overall survival in different modules.


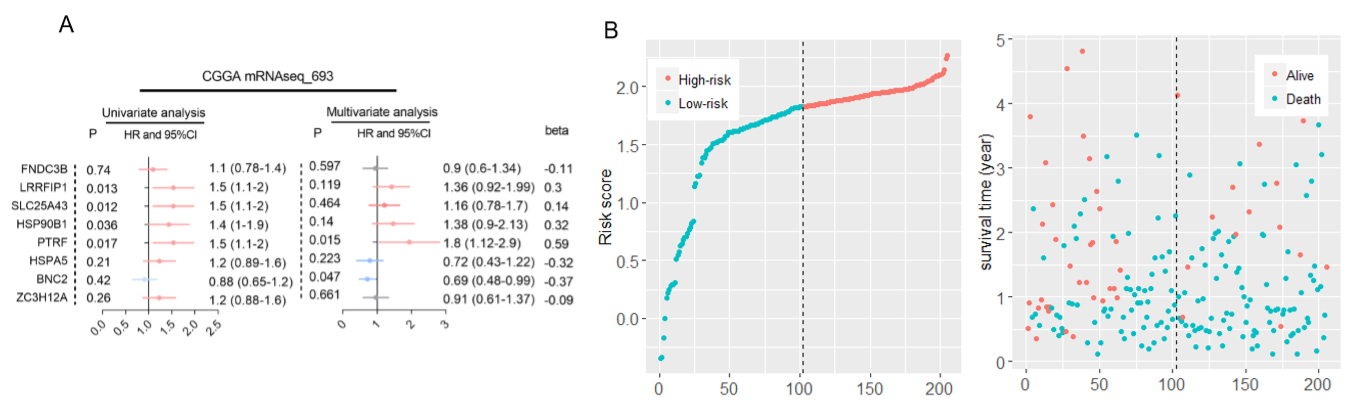


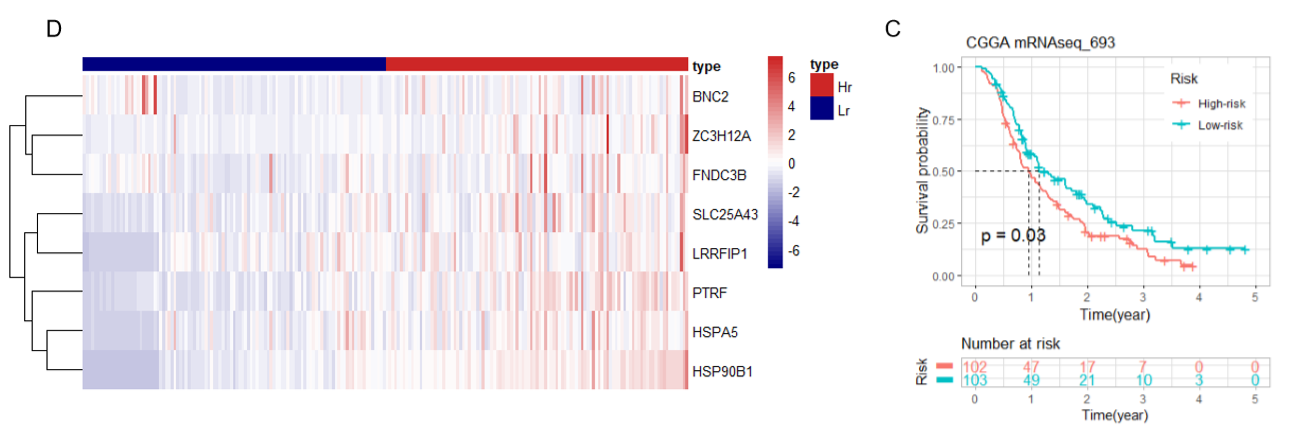


**Figure S4.** Survival analysis verification of prognostic gene signature of the eight RBPs in the GBM patients (CGGA mRNAseq_693 dataset). (**A**) Univariate and multivariate survival analysis in CGGA mRNAseq_693 dataset. (**B**) The risk score distribution (left) and survival status distribution (right) in 205 GBM patients. (**C**) The Kaplan–Meier survival curves for high- and low-risk groups. (**D**) The heat map of eight RBPs for high- and low-risk groups .


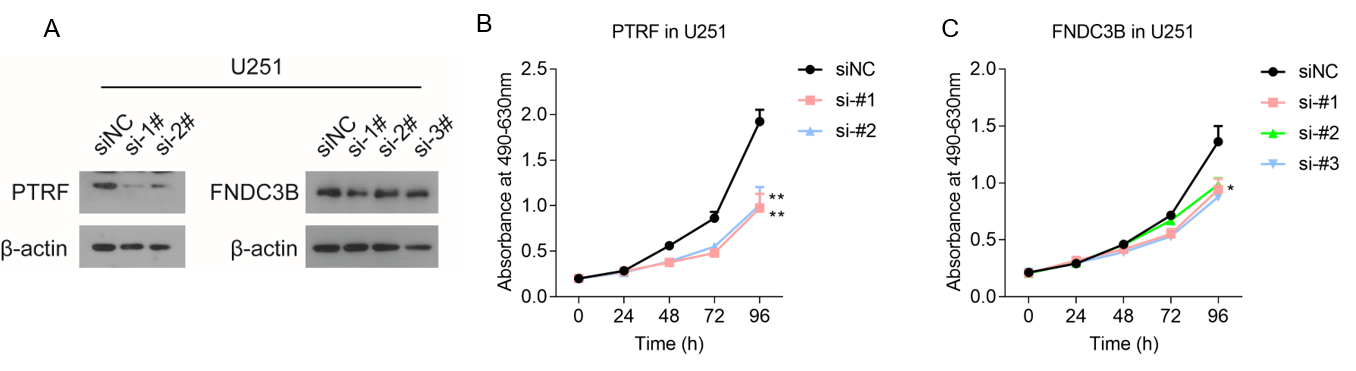


**Figure S5.** Detection of cell proliferation ability in U251 cell. (**A**) western blot to detect knockdown effect of PTRF and FNDC3B in U251 cell.(**B**) Cell Proliferation Assay after knock down of HSP90B1 in LN229. (**C**) Cell Proliferation Assay after knockdown of FNDC3B in U251. (* *p* < 0.05, ** *p* < 0.01)


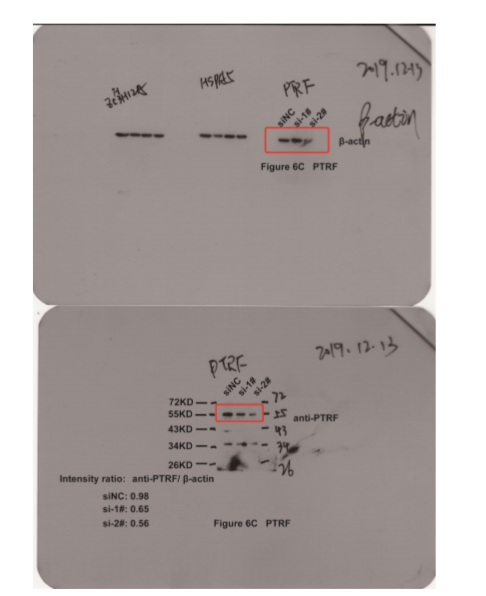

A


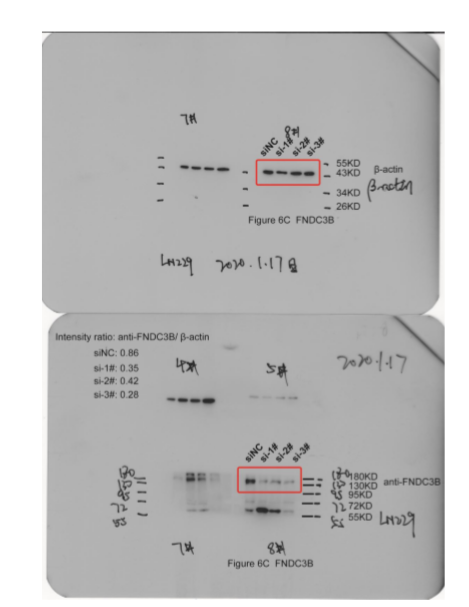


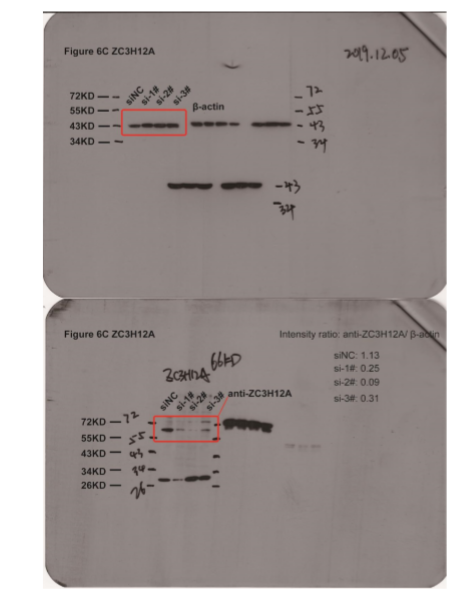


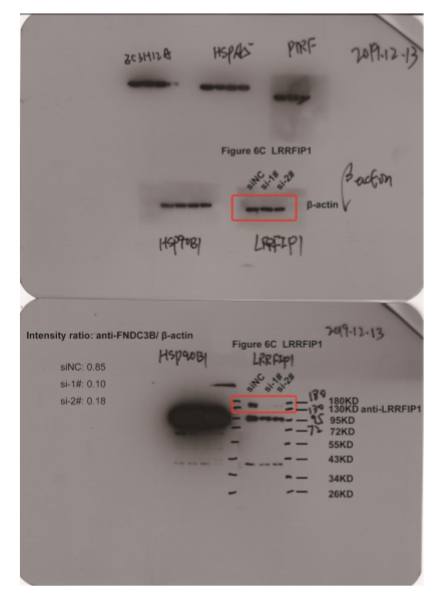


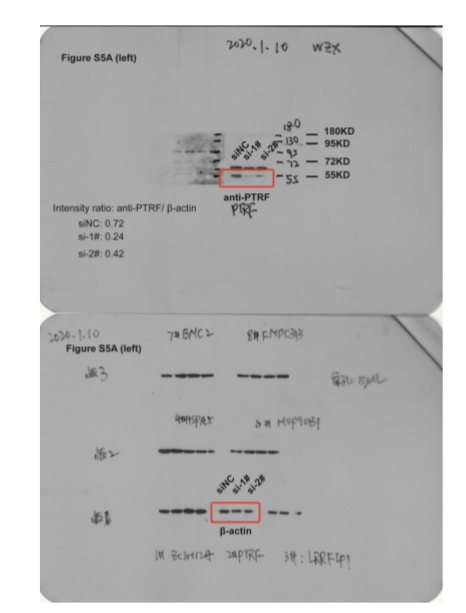
B


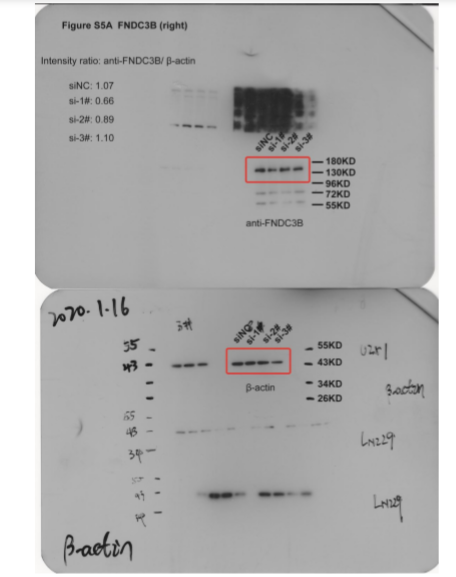


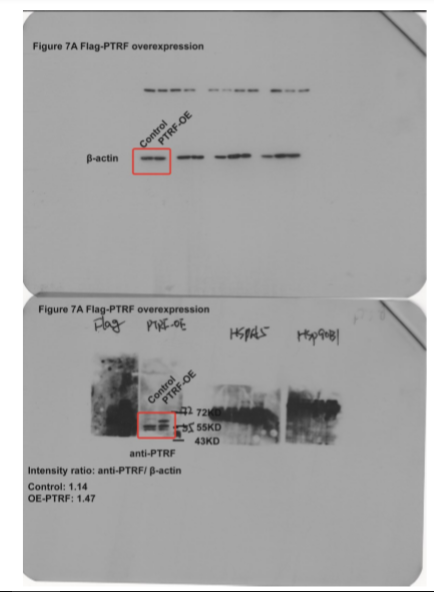
C


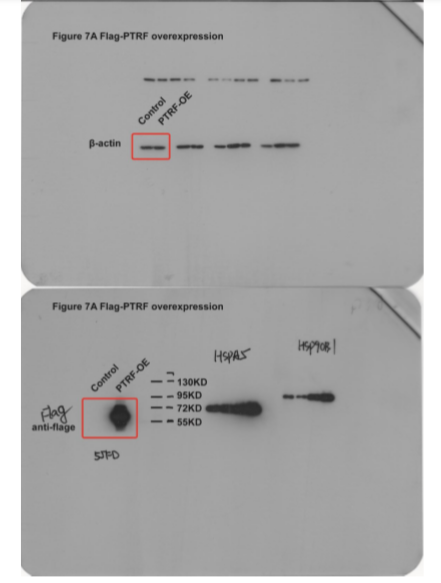


Figure S6 The uncropped western blot figures were shown with intensity ratio. (**A**) The uncropped western blot used to detect the knockdown effect of PTRF, FNDC3B, ZC3H12A, and LRRFIP1 related to figure 6C, (**B**) The uncropped western blot to detect knockdown effect of PTRF and FNDC3B in U251 cell related to Figure S5A, (**C**) The uncropped western blot confirming the expression of PTRF in the Flag-PTRF overexpression’s LN229 cell related to Figure 7A.

| 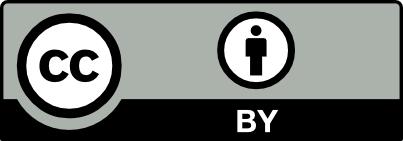 | © 2020 by the authors. Licensee MDPI, Basel, Switzerland. This article is an open access article distributed under the terms and conditions of the Creative Commons Attribution (CC BY) license (http://creativecommons.org/licenses/by/4.0/). |
| --- | --- |
